# Early detection of Blunt traumatic brachial artery pseudoaneurysm by point of care ultrasound: case report

**DOI:** 10.1186/2036-7902-7-S1-A29

**Published:** 2015-03-09

**Authors:** Jen-Tang Sun, Ming-TseJ Tsai, Chun-Yen Huang, Hong-Wei Chen, Kuang-Chau Tsai, Wan-Ching Lien, Hsiu-po Wang

**Affiliations:** 1 Department of Emergency Medicine, Far Eastern Memorial Hospital, New Taipei City, Taiwan; 2 Department of Emergency Medicine, National Taiwan University Hospital, Taipei City, Taiwan; 3 Department of Internal Medicine, National Taiwan University Hospital, Taipei City, Taiwan

**Keywords:** Brachial Artery, Humeral Fracture, Ulnar Artery, Artery Pseudoaneurysm, Blunt Injury

## Background

Traumatic pseudoaneurysm(PA) most commonly with penetrating injury, Blunt mechanism is very rare and often delay diagnosis.We present one case of blunt injury induced brachial artery PA diagnosed by point of care ultrasound(US).

## Patient and method

**Figure 1:**
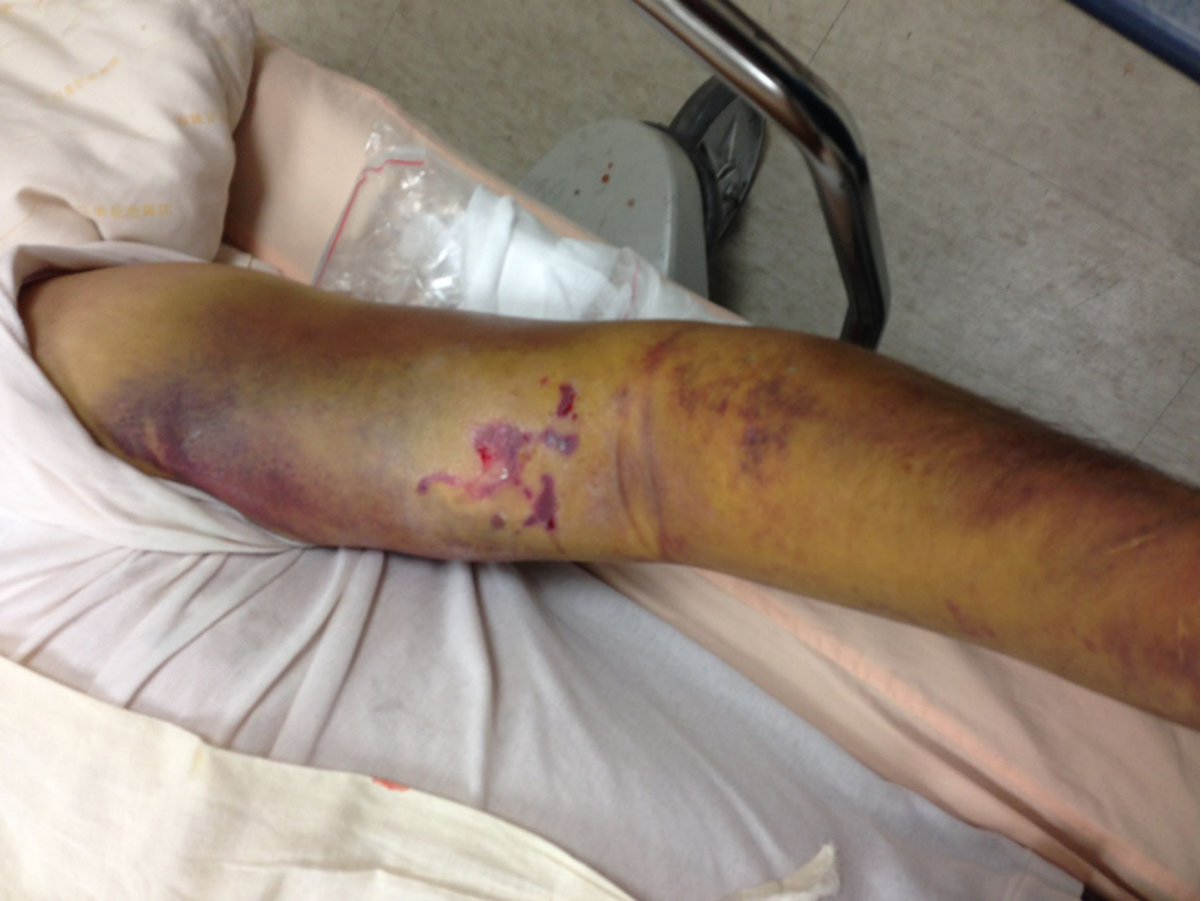
Figure 1

**Figure 2:**
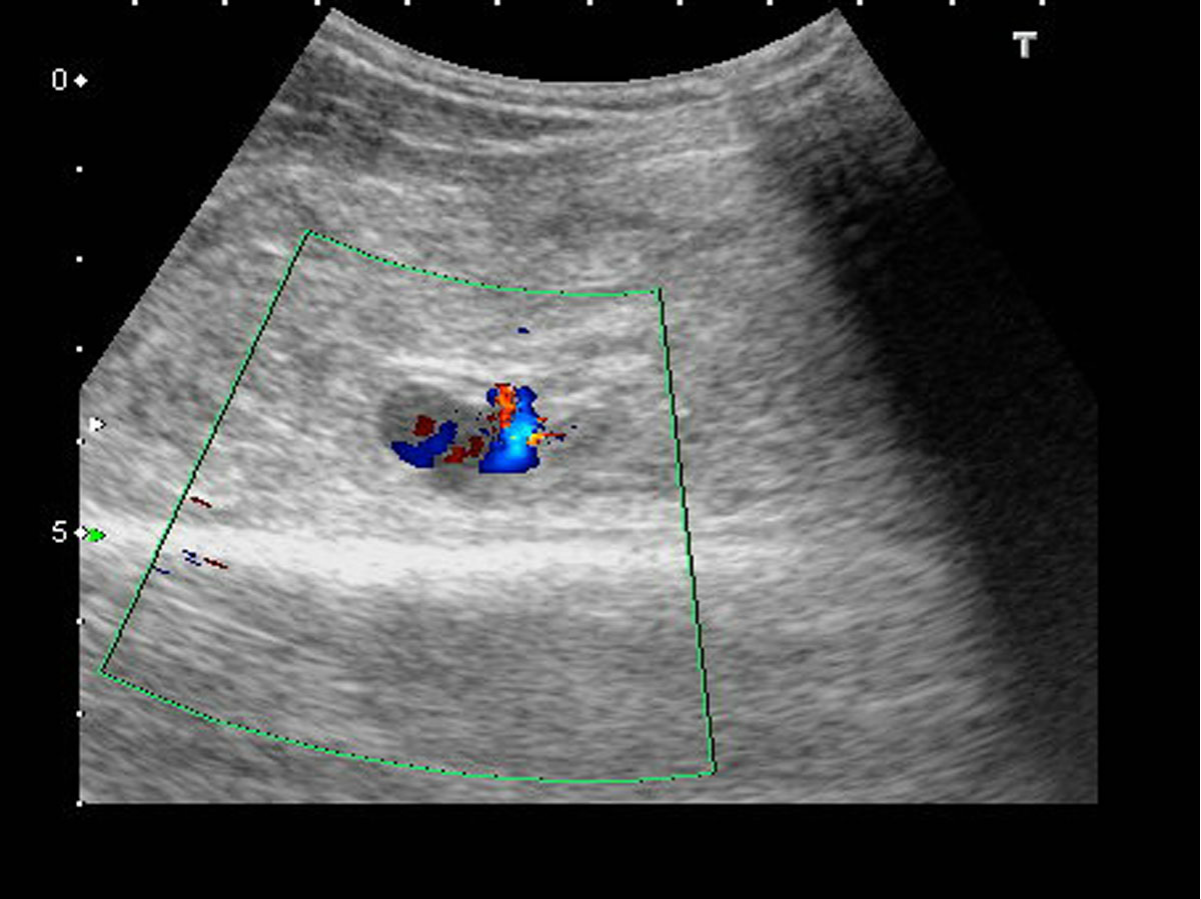
Figure 2

**Figure 3:**
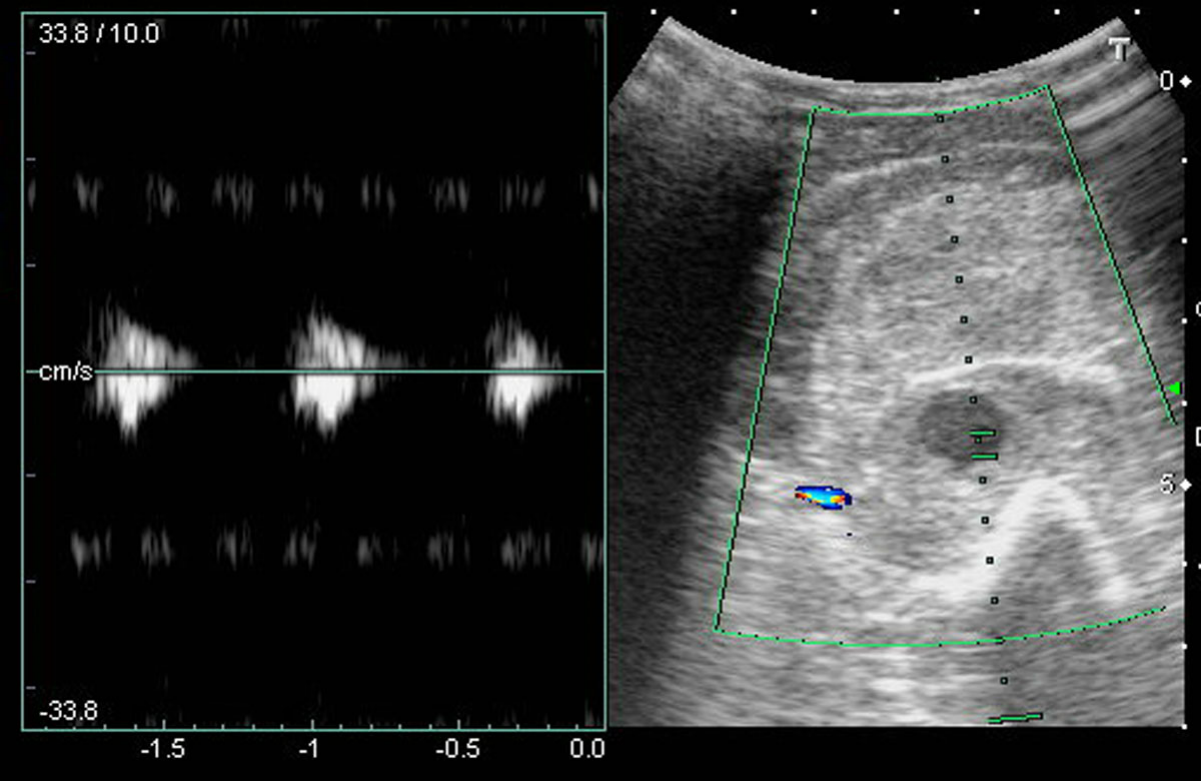
Figure 3

**Figure 4:**
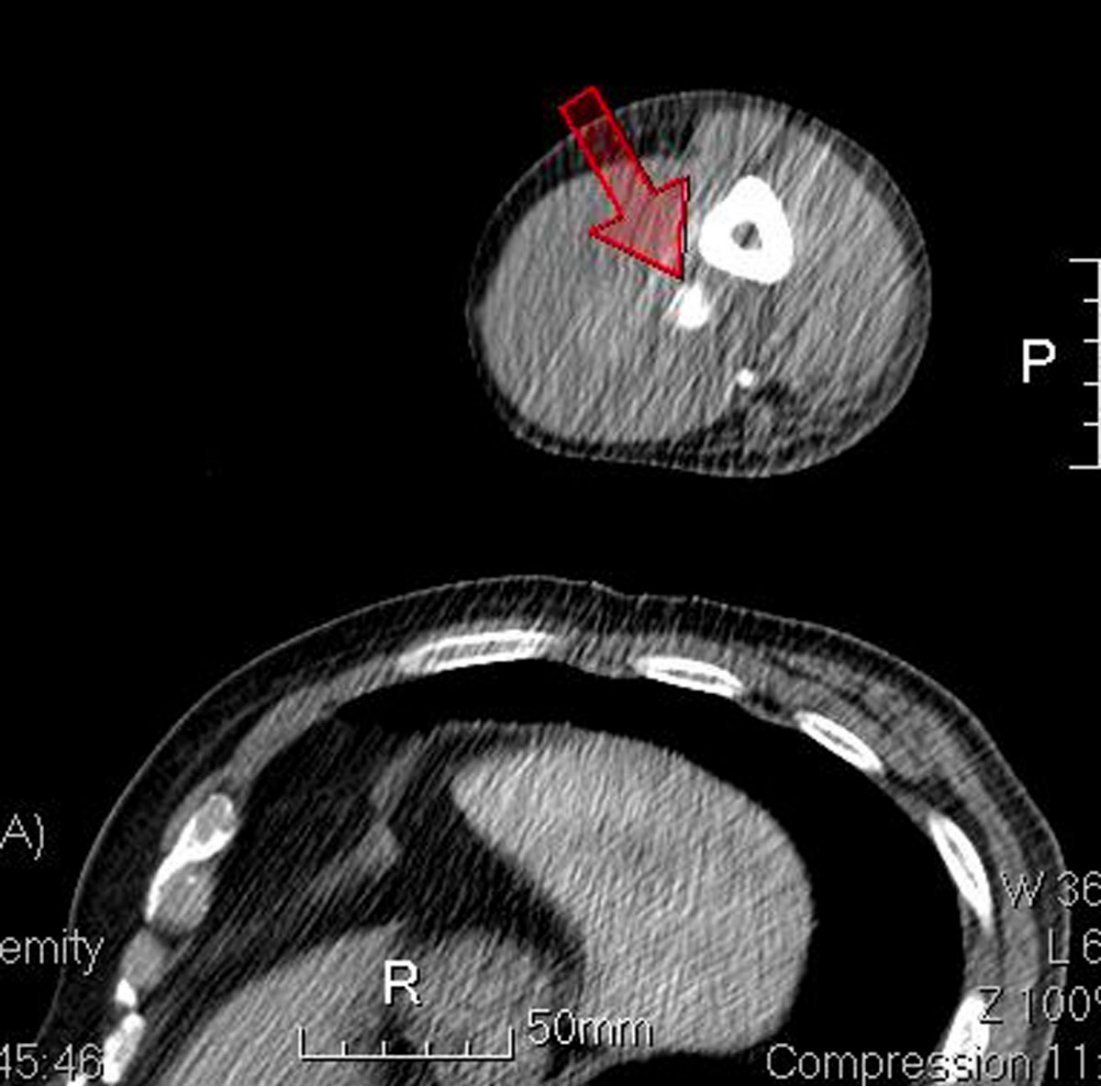
Figure 4

**Figure 5:**
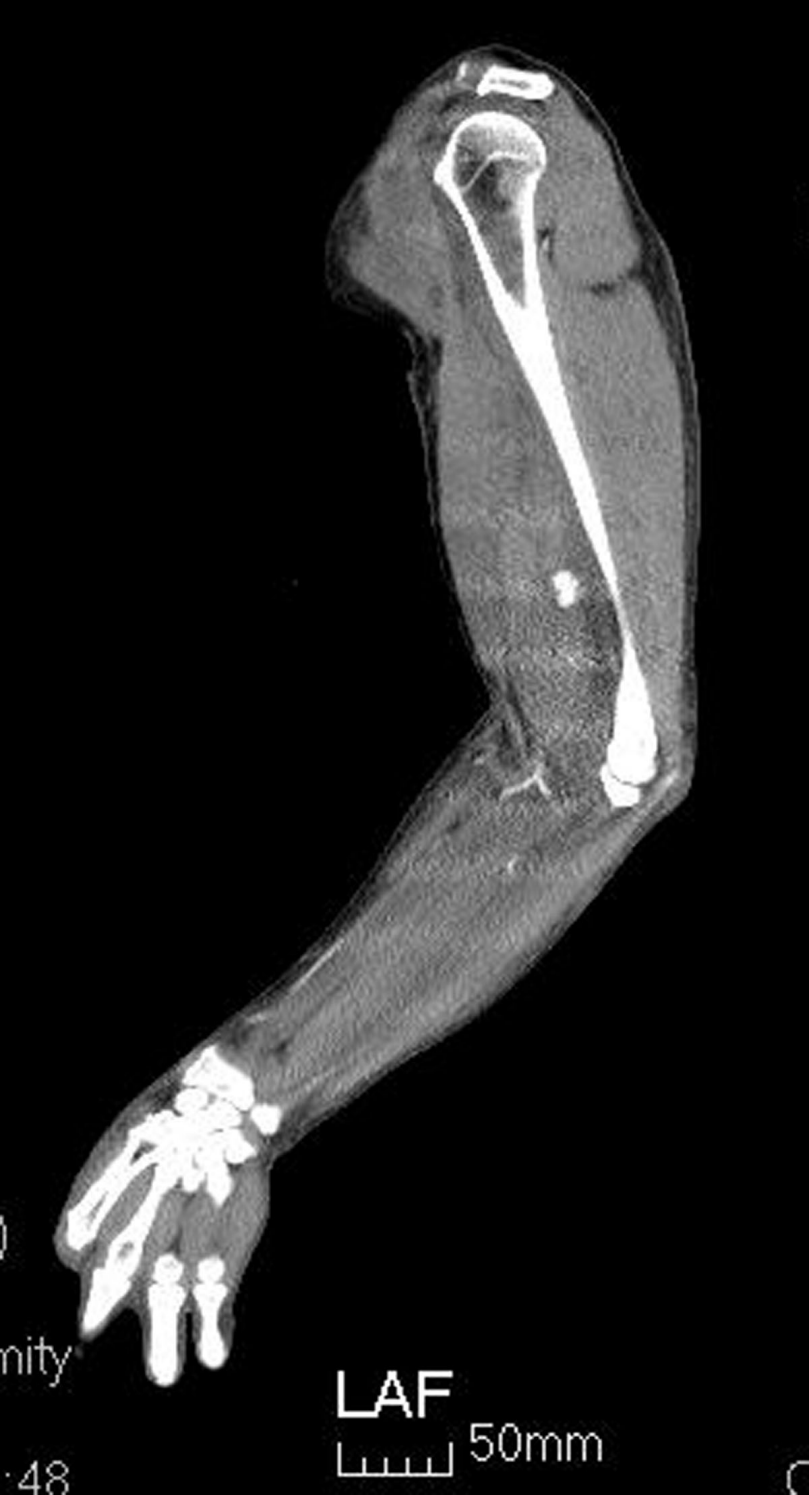
Figure 5

A 61-year-old man with hypertension history, presented with progressive left elbow pain and swelling after blunt injury. He was fell down 1 week ago and direct contusion to his arm. He reported no numbness or weakness. His vital signs were stable except high blood pressure (188/108mHg) Physical examination revealed ecchymosis of his arm (Figure 1) and palpable pulsation over brachial, radial and ulnar artery. Laboratory exam showed elevatingCK(793 IU/L) otherwise normal. X-ray exam revealed normal. US revealed an anechoiclesion over elbow with pulsation.(Figures 2 and 3) and some heterogenic lesion over muscle layer, PA and hematoma were considered. CTA of extremity showed PA of brachial artery.(Figures 4 and 5) Patient received endografting and fasciotomy, patient was discharged smoothly after 10 days admission.

## Result

Brachial artery PA can be from 3 etiology:congenital, systemic disease, and trauma. Penetrating injury is most common mechanism.1 Clinical symptoms including redness, induration/ pulsatile mass and pain, Most case of brachial artery PA has delay presentation. In the literature review only 1 case was found for blunt traumatic mechanism not associated without humeral fracture,Inthese two cases were all diagnosed by US. Duplex US has good sensitivity and specificity in postcatheterizationpatients[3] Typical US finding including cystic lesion adjacent to a supplying artery, ying-yang sign in cystic mass, to end fro sign.[4] US also can be used in treatment, like ultrasound guide thrombin injection. Other diagnostic tools including CTA or angiography, but all have radiation.

## Conclusion

Although PA of brachial artery in blunt trauma is rare disease, but should be considered in delay presentation after trauma. Point of care US can properly diagnose the PA in ED setting.

## Informed consent

The study was conducted in accordance with the ethical standards dictated by applicable law. Informed consent was obtained from each owner to enrolment in the study and to the inclusion in this article of information that could potentially lead to their identification.
